# Aluminium Phosphide Poisoning: Early Suspicion of Cardiotoxicity Is Necessary for Improved Outcomes

**DOI:** 10.7759/cureus.10237

**Published:** 2020-09-04

**Authors:** Debananda Sahoo, Shiny T Kujur, Dhriti Sundar Das, Anupam Dey, Sujata Devi

**Affiliations:** 1 General Medicine, All India Institute of Medical Sciences, Bhubaneswar, IND; 2 General Medicine, Kalinga Institute of Medical Sciences, Bhubaneswar, IND

**Keywords:** aluminium phosphide, suicide, phosphene gas, rodenticide, regional wall motion abnormalities

## Abstract

Poisoning is one of the more conventional modes of suicide in some parts of India. Aluminium phosphide (ALP) is a chemical used for this purpose and manifests severe cardiovascular complications, such as hypotension, shock, various arrhythmias, congestive heart failure with toxic myocarditis, and in rare cases, ST-segment elevation myocardial infarction or other electrocardiogram changes. Upon contact with moisture, ALP yields phosphine gas, a toxic systemic poison found in pesticides that can lead to cardiovascular-related mortality. We present a case of ALP poisoning in a 60-year-old woman who was asymptomatic for the first 48 hours. She gradually developed cardiac complications in the form of anteroseptal acute myocardial infarction (AMI). As AMI is very rare among the various cardiac complications, an early vigilance is necessary to prevent further complications in ALP poisoning.

## Introduction

Suicide is among the 10 leading causes of death in the world and accounts for more than 400,000 deaths annually [1]. Poisoning is one of the more conventional methods adopted for suicide. Substances used for poisoning may vary in different parts of the world, depending largely on availability and access to the substances [1,2].

Aluminium phosphide (ALP) is a highly effective rodenticide used in India and throughout the world to protect stored rice and other grains from rodents. The ALP tablet used to deter rodents is extremely lethal: even 1/6 (500 mg) of an unexpired tablet may be fatal if consumed.

Although almost all organ systems are affected, the respiratory, cardiovascular, and nervous systems are the most critically affected. Cardiovascular arrhythmias with non-specific ST-T changes have been seen on an electrocardiogram (ECG) but can be present in myocardial depression with a refractory shock, which can be fatal. We report a rare case of ALP poisoning with myocardial depression seen on ECG as an anteroseptal acute myocardial infarction (AMI).

## Case presentation

A 60-year-old woman presented to our emergency department (ED) with a stated history of consumption of celphos (ALP) mixed with water having a suicidal intention about 10 hours before she arrived at our facility. She was unsure about the quantity of ALP she ingested.

**Figure 1:**
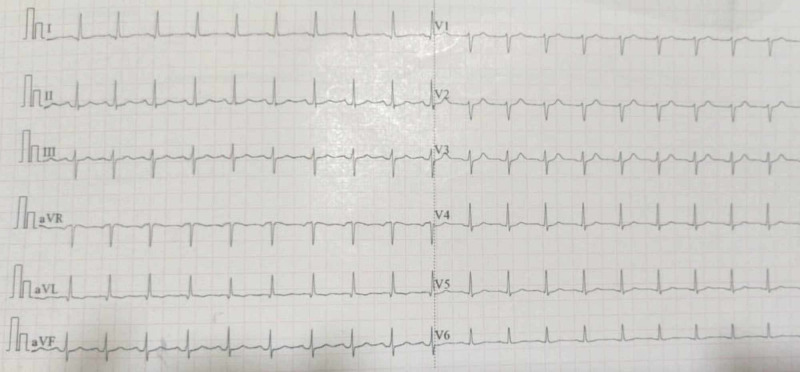
Electrocardiogram changes in the present case on day 1

She reported four to five vomiting episodes that occurred before reaching the hospital. At the ED, her vitals were as follows: pulse rate was 108/minute, oxygen saturation was 98% on room air, and blood pressure was 124/80 mmHg. The patient was conscious and oriented. Her Glasgow Coma Scale score was E4V5M6. Additional findings included clear chest cavity sounds with S1 and S2 normally audible without any murmur. Her abdomen was soft and non-tender with normal bowel sounds, and she demonstrated bilateral plantar flexion and pupil reactions to light that were within normal limits. Laboratory and procedural tests requested upon her admission to the ED demonstrated normal ECG (Figure 1), random blood glucose test, and troponin-I results.

Additional blood test results were as follows: her complete blood count total leucocyte count was 6.59 × 10^3^/mm^3^ (4-11 × 10^3^/mm^3^), her haemoglobin was 10.4 g/dl (12-16 g/dl), total platelet count was 156 × 10^3^/mm^3 ^(150-450 × 10^3^/mm^3^), serum urea/creatinine was 46/1.4 mg/dl (15-40/0.5-1.2 mg/dl), serum sodium/potassium was 140/4.1 mEq/l (135-145/3.5-5 mEq/l), and aspartate transaminase/alanine aminotransferase was 32/33 U/L (12-38/7-41 U/L). The patient was admitted for close monitoring of anticipated systemic complications and treated symptomatically with intravenous pantoprazole, ondansetron, magnesium sulphate, and fluids. The patient was advised to rest. The first two days following admission were unremarkable and did not involve any clinical or biochemical deterioration.

**Figure 2:**
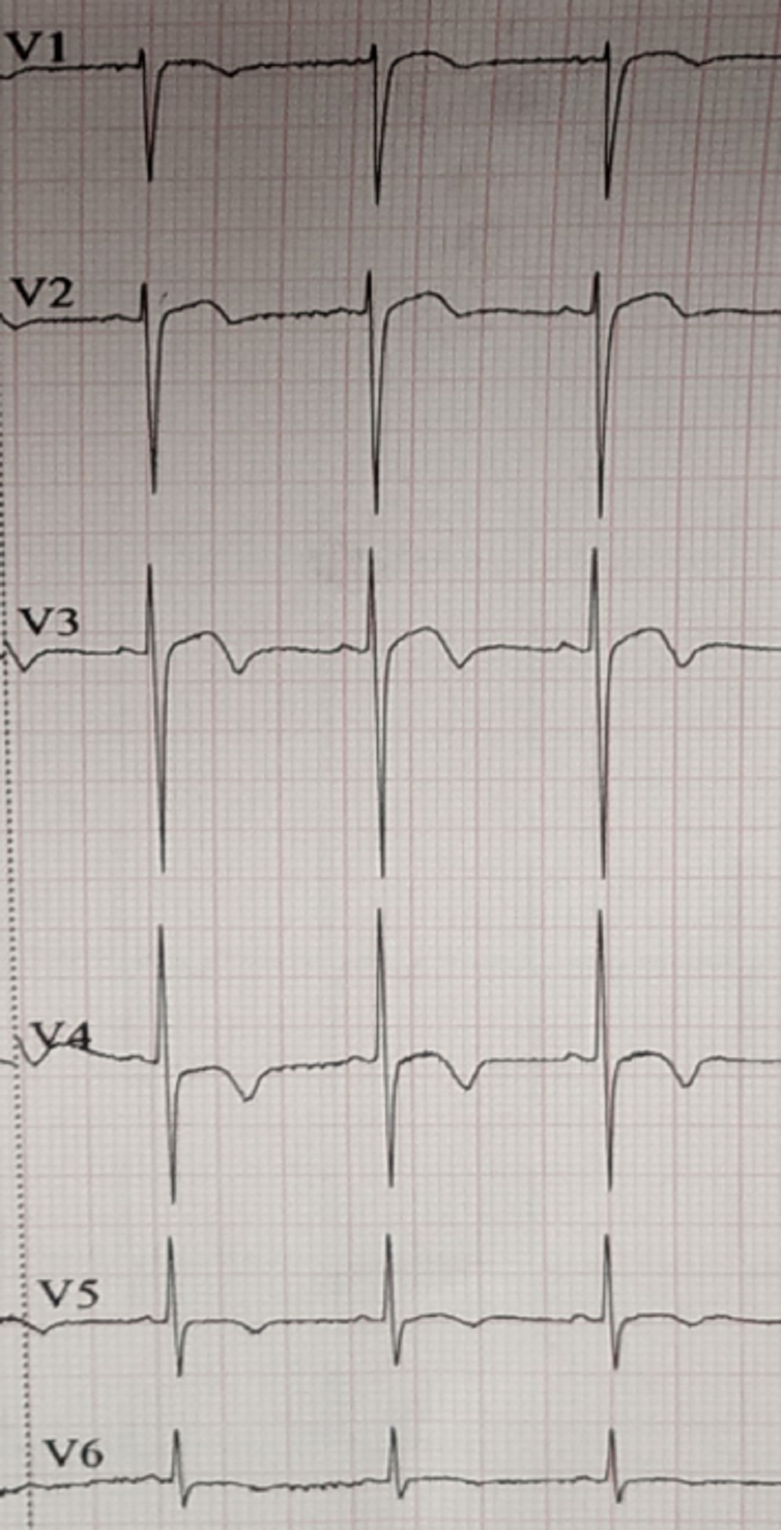
Electrocardiogram changes in the present case on day 3

On day 3, the patient was symptomatic with shortness of breath on exertion, and her ECG showed diffuse ST and T changes, specifically ST elevation with coving pattern in leads V2 and V3 and associated T inversion from leads V2 to V6 (Figure 2).

A two-dimensional (2D) echocardiogram (ECHO) was immediately conducted, which showed there was no regional wall motion abnormality and an ejection fraction of 62% that is within the normal range (55%-70%). Arterial blood gas, sodium, potassium, and thyroid-stimulating hormone test results were also within reference ranges. The patient’s creatinine phosphokinase-myocardial band result was considered high at 233 IU/L (26-192 IU/L). Her troponin-I test results of two blood samples drawn at six hour apart were within the reference range (0.00-0.40 ng/ml). As the patient refused for any interventional therapy, she was started on medications for the acute coronary syndrome in the form of low molecular weight heparin, aspirin, clopidogrel, atorvastatin, and metoprolol as per the opinion of the cardiologist.

On days 4 through 6 of admission, the patient was mildly symptomatic with persisting ECG changes (T inversion, V2-V6).

**Figure 3:**
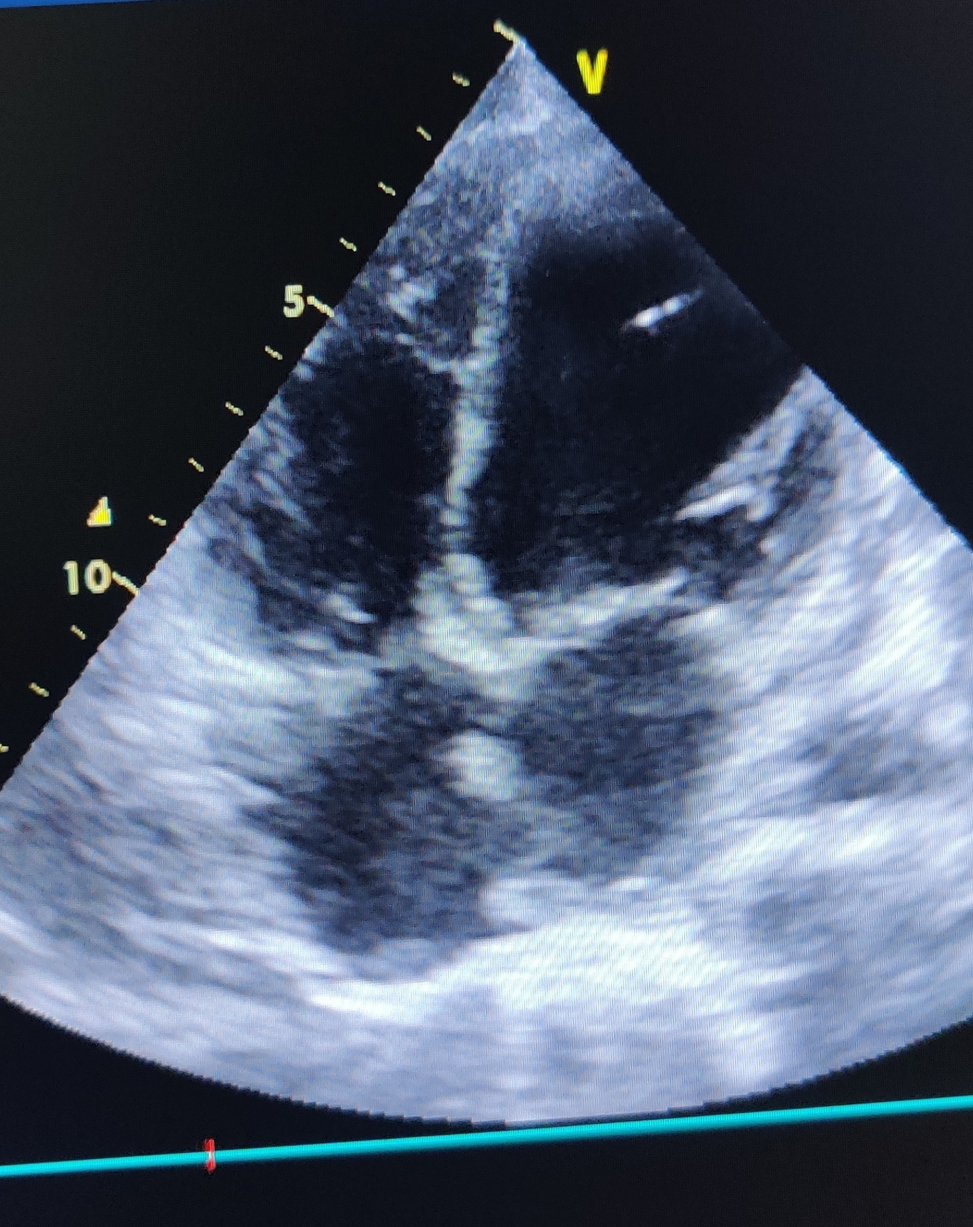
Repeat 2D echocardiogram (ECHO) in the present case on day 7

The patient was asymptomatic on day 7. Due to the persistence of ECG changes, a qualitative troponin-I level test was repeated, the results of which were positive. A repeat 2D ECHO demonstrated regional wall motion abnormality in the left anterior descending (LAD) coronary artery territory, an ejection fraction measurement of 45%, mild left ventricular systolic dysfunction, mild hypokinesia of the anterior wall, and a distal interventricular septum (Figure 3).

A coronary angiogram was planned but refused by the patient. 

As her vitals were stable and the patient was asymptomatic, she was discharged with medications, including aspirin (75 mg), atorvastatin (40 mg), clopidogrel (75 mg), metoprolol (12.5 mg), and sorbitrate tablets (5 mg). Before discharging, psychiatric consultation was taken to address her behavioural issues suggestive of deliberate self-harm and was advised for regular psychiatry outpatient department review. She was advised to avoid strenuous physical activity and asked to follow up later with the outpatient department regularly.

After one month, the patient was reviewed by the outpatient department. She was asymptomatic and doing well. Upon repeat ECHO, regional wall motion abnormalities persisted with mild improvement in ejection fraction, which had increased to 52%.

## Discussion

ALP is used as a rodenticide and is commonly used as a suicidal agent in some parts of India [3]. The classic presentation of ALP poisoning is epigastric pain, nausea, and cardiogenic shock reflected as severe refractory hypotension, as described in many case reports [4].

Upon contact with moisture in the environment, ALP undergoes a chemical reaction yielding phosphine gas, which is an active pesticidal component and a very toxic systemic poison [5]. At the cellular level, phosphine inhibits mitochondrial oxidation of cytochrome C and impairs cellular inspiration. This leads to anaerobic respiration replacing aerobic respiration and subsequently high levels of lactic acid, progressive refractory metabolic acidosis, and profound circulatory collapse [6,7]. The clinical course of ALP poisoning usually starts with nausea and vomiting and proceeds to multiorgan failure and death within 24 to 48 hours after poisoning [8,9].

The primary clinical manifestation reported in literature comprises of the cardiovascular system (60%-100%), including shock and cardiac arrhythmias as prominent features [3].

Cardiovascular involvement is common in ALP poisoning and is manifested by hypotension, shock, bradycardia or tachycardia arrhythmia, congestive heart failure with toxic myocarditis, and ECG abnormalities. ECG changes related to celphos poisoning have been examined in various studies and include atrial fibrillation, supraventricular and ventricular tachycardia, ST-T changes, bundle branch blocks, and atrioventricular conduction disturbances. Echocardiographic findings include decreased ejection fraction, generalized hypokinesia of the left ventricle, and pericardial effusion [10,11]. However, regional wall motion abnormalities along an arterial territory with AMI presentation are exceptionally uncommon. Usually, ALP poisoning presented with various cardiac complications and ECG changes within first 24 hours of poisoning [12]. However, in the present case, without any previous history of cardiovascular abnormality or any cardiovascular risk factor except age, the patient demonstrated an elevated ST-segment with the T-wave inversion after 48 hours of admission. Her 2D ECHO findings were within reference ranges initially, but upon repeat testing one week after admission, the findings demonstrated regional wall motion abnormality in the LAD territory. All these may be due to delayed cardiac complications of ALP poisoning which cannot be explained at this juncture with a single case but maybe in future with a more extensive study.

## Conclusions

ALP tablet poisoning is associated with very high mortality, primarily due to circulatory collapse. Most patients with ALP poisoning develop cardiac arrhythmias, which are invariably life-threatening, as well as ECG changes of ST depression and bundle branch block. ECHO usually shows global hypokinesia with depressed ejection fraction. As reported in our case, a regional wall motion abnormality is a rare phenomenon associated with this condition. Therefore, an early vigilance for cardiac complications with an eye on delayed cardiac complications is necessary to prevent further complications resulting from ALP poisoning.
